# Flexible Electromagnetic Shielding Nano-Composites Based on Silicon and NiFe_2_O_4_ Powders

**DOI:** 10.3390/polym15112447

**Published:** 2023-05-25

**Authors:** Alina R. Caramitu, Romeo C. Ciobanu, Ioana Ion, Cristina M. Schreiner, Mihaela Aradoaei, Violeta Tsakiris, Jana Pintea, Virgil Marinescu

**Affiliations:** 1 National Institute for Research and Development in Electrical Engineering ICPE—CA, 030138 Bucharest, Romania; alina.caramitu@icpe-ca.ro (A.R.C.); ioana.ion@icpe-ca.ro (I.I.); violeta.tsakiris@icpe-ca.ro (V.T.); jana.pintea@icpe-ca.ro (J.P.); virgil.marinescu@icpe-ca.ro (V.M.); 2 Department of Electrical Measurements and Materials, Gheorghe Asachi Technical University, 700050 Iasi, Romania; cristina-mihaela.schreiner@academic.tuiasi.ro (C.M.S.); mihaela.aradoaei@academic.tuiasi.ro (M.A.)

**Keywords:** flexible electromagnetic shielding, silicon composites with NiFe_2_O_4_ nano-powders, dielectric characteristics, electromagnetic shielding effectiveness, thermal stability, lifetime

## Abstract

In this paper, the obtaining and characterization of five experimental models of novel polymer composite materials with ferrite nano-powder are presented. The composites were obtained by mechanically mixing two components and pressing the obtained mixture on a hot plate press. The ferrite powders were obtained by an innovative economic co-precipitation route. The characterization of these composites consisted of physical and thermal properties: hydrostatic density, scanning electron microscopy (SEM), and TG DSC thermal analyses, along with functional electromagnetic tests in order to demonstrate the functionality of these materials as electromagnetic shields (magnetic permeability, dielectric characteristics, and shielding effectiveness). The purpose of this work was to obtain a flexible composite material, applicable to any type of architecture for the electrical and automotive industry, necessary for protection against electromagnetic interference. The results demonstrated the efficiency of such materials at lower frequencies, but also in the microwave domain, with higher thermal stability and lifetime.

## 1. Introduction

Composites are widely investigated in recent years in order to discover new developments for an optimal living and working environment, and different performances are tailored, depending on the desired application [1]. The continuous increase in electricity production, due to the ever-increasing demand of consumers, which generates electromagnetic fields, leads to an increase in the electromagnetic pollution of the environment in various rooms/spaces/offices [2]. Electromagnetic pollution, continuously increasing, can even lead to the deterioration of human health [3,4,5,6] and living matter [7,8,9,10,11,12,13,14]. Reducing the electromagnetic pollution created in different spaces with different uses can be achieved by researching and designing different new materials that are effective for this purpose [15,16]. 

Low-frequency electromagnetic shielding is the technique in which low-frequency signals are blocked from interfering with the source circuit as well as the neighboring circuits by using enclosures commonly made of conductive materials. Compared to high-frequency electromagnetic fields, low-frequency electromagnetic fields are harder to prevent from reaching sensitive circuits. As the frequency of electromagnetic waves decreases, the field strength increases, and reflection and absorption losses decrease. If designing electromagnetic filters for low-frequency electromagnetic field mitigation, then the size of the inductors involved will be large. Considering all these factors, special shielding techniques for low-frequency electromagnetic fields are required. The materials chosen are usually of high permeability so that they can divert electromagnetic fields from the devices of interest. Low-frequency electromagnetic field shielding is effective in reducing the electromagnetic field strength by around 95%. The strength of the electric and magnetic fields is high at low frequencies, so highly permeable materials are used for low-frequency electromagnetic field shielding. The selection of materials depends on the frequency and the strength of the electromagnetic fields needing to be blocked. The typical materials used for electromagnetic shielding are copper and aluminum. Highly permeable materials with low reluctance, such as steel, are also particularly effective in low-frequency shielding. As long as the use of metallic shielding can create difficulties in electronic devices assembling in different industries, composite materials are tested as potential candidates for electromagnetic shielding at low frequencies. When using non-ferromagnetic-based composite materials, time-varying magnetic flux determines an induced electric field in the shield material, producing an induced electric field, which will generate a magnetic field opposite to the field from the outside. The shielding factor depends only on the electric conductivity of the shield material. In the case of ferromagnetic-based composite materials, the shielding factor of the shield depends on the penetration depth of the magnetic field, so depends on the permeability and electric conductivity of the material.

In both cases, the electromagnetic shielding efficiency (SE) of composites must be at least 20 dB to meet the minimum standard for commercial applications; consequently, the conductivity of the material must be about 1 S/m [17,18,19,20,21]. The addition of conductive or ferrite fillers in a diverse polymer matrix is a common procedure towards achieving specialized features, e.g., epsilon-near-zero properties [22], negative permittivity [23], or thermal management [24], but the achieving of high electromagnetic shielding at lower frequencies remains a challenge when trying to substitute the thicker materials containing important quantities of metallic fillers, e.g., [25,26,27], even if with some addition of ferrites in some cases. In the commercial composites used for electromagnetic shielding, the fillers are randomly distributed in the polymer matrix, resulting in weak overlaps, and these composites can be said not to have reached percolation. In order to reach percolation, it is absolutely necessary to add high concentrations of filler to the polymer matrix [28,29,30,31]. Conductive paths can thus be created leading to effective shielding. However, this not only significantly increases the manufacturing costs and the difficulty of processing the materials, but also induces a high density which leads to the limitation of their use in certain applications. Thus, obtaining composite materials with well-defined conductive networks is one of the most effective methods of obtaining high-performance electromagnetic shielding materials.

Recent studies have demonstrated that the major increase in the concentration of magnetic fillers in the composition of the polymer composite material, but also an important addition of conductive particles such as metallic or CNT, improves the shielding capacity/efficiency and at the same time avoids the interface effects by creating an external magnetic field [17,20,32].

To improve the conductivity of composites at low filler concentrations, layered composites are often used to promote selective dispersion of the filler material and increase their efficiency. However, many of these composites show defects at the matrix-filler interface, which affects their mechanical properties [21,33,34,35,36]. Currently, the reflection of EM waves is the primary shielding mechanism in most composites with shielding uses, leading to serious secondary EM radiation pollution. Thus, it is very important for composite materials to exhibit low densities, high shielding efficiencies, and good mechanical properties without excessive secondary EM radiation.

These materials must simultaneously fulfill certain conditions. It must ensure an acceptable attenuation of electromagnetic waves and at the same time, it must present high mechanical performances for low mass, high resistance to UV radiation, resistance to the action of mold, etc. These performances can be ensured by polymer-based composite materials, which have good mechanical properties [37], good thermal stability [38,39,40], high resistance to the action of mold [10,39,41], UV resistance [42], low hydrophilicity [43], etc. The fillers used to make composite materials for attenuation of electromagnetic waves are metals [44], and carbonic or ferrite powders. Ferrite particles have improved performance at sizes below 30 nm [45]. Thus, these polymer nanocomposite materials doped with ferrite, have exceptional shielding properties [46]. Ferrite particles have a decisive role in various fields of science and technology due to their absorptive and catalytic nature, high stability to thermal, chemical and mechanical stress, tunable chemical composition, but also controllable magnetic properties [47].

Due to the advantages presented by a flexible matrix, many researchers have used silicon or natural rubber as the matrix in composite materials for electromagnetic attenuation applications. Thus, Raheem A. Jabur [48] obtained a composite of silicon and ferrite of Cr and Mn with applications in microwave absorption in the 8-12 GHz range. Hemeda O.M. [49] made a silicone rubber/NiCr_0.2_Fe_1.8_O composite material to obtain permanent magnets. Most of the research carried out was aimed at diversifying the performance of permanent magnets, due to ferrites characteristics such as low melting point, high specific heating, large expansion coefficient, low saturation magnetic moment, and low magnetic transition temperature.

In this work, polymer composite materials of silicon with nickel ferrite fillers were obtained and tested for application as electromagnetic shields for low frequencies. NiFe_2_O_4_, a soft super-paramagnetic material with magnetic semiconductor features, was peculiarly chosen, for the purpose of avoiding the supplementary addition of conductive powders, a procedure commonly used to improve the ferrite behavior under the electromagnetic field. NiFe_2_O_4_ is one of the representants of the most attractive class of ferrite materials due to its interesting properties with up-to-date technical applications, such as gas-sensor, magnetic fluids, catalysts, magnetic storage systems, photomagnetic materials, magnetic resonance imaging, site-specific drug delivery, and microwave devices. Even if some very recent papers addressed the subject of composites from rubber and ferrites, they are analyzing other types of rubbers and completely other types of ferrites, e.g., manganese–zinc, or nickel–zinc [27]. The silicone rubber class presents characteristics of both inorganic and organic materials and offers a number of advantages that are not found in the organic rubbers. Silicone rubbers have good chemical stability and flame retardancy, and superior resistance to heat and cold. Based on nickel ferrite features, the composite materials of silicon with nickel ferrite fillers may exhibit useful properties in the domain of electromagnetic shielding at lower frequencies, offering also complementary advantages, i.e., lower thickness for the same shielding efficiency, compared to similar composites, and flexibility, a feature with great potential in automotive and flexible electronics domains. On the other hand, another important advantage lies in the possibility of making multi-layer structures or linked structures, including by use of common commercial silicon adhesives, which maintain the flexibility of the structure, an advantage not met by other types of rubbers, where no compatible adhesives are available to maintain both flexibility and strength. 

No complete study was addressing the manufacturing and testing of such composites up to now, even if some papers addressed the properties of, e.g., natural rubber composites with other types of nickel ferrites [50], and so the innovative concept presented in the paper may bring clear benefits for the users. The characterization of these composite materials consisted of the determination of the hydrostatic density, SEM analyses, TG DSC analyses, and finally, dedicated tests to demonstrate the functionality of such materials as electromagnetic shields with proven flexibility, namely by the determination of the magnetic permeability, the dielectric characteristics, as well as the determination of the efficiency of electromagnetic shielding and lifetime.

## 2. Materials and Preparation Methods

### 2.1. Materials

The following raw materials were used to obtain the 5 types of composite materials:

- Bi-component silicone rubber with the commercial name HT 45 addition, with properties as in Table 1 [51].

- The ferrite powder was synthesized by a simple and innovative co-precipitation route, mixing suitable volumes of 10 mM solution of Fe^3+^ and 10 mM Ni^2+^ solution, in order to obtain a molar ratio of 2:1. The solution was titrated with an excess of NaOH at 90 °C, and stirred for 30 min. The obtained precipitate was washed with distilled water three times and dried. In order to increase the crystallinity degree of ferrite powders annealed at 700 °C for 3 h. The average dimension of synthesized particles was about 750 nm. The procedure is much simpler and economical compared to similar ones, co-precipitation with mechanical alloying, or sol–gel auto-combustion, as in [52,53,54], but the respective ones are destinated for achieving particles under 50 nm, very useful for other applications, as permanent magnets. However, in our case, particles with sub-micron dimensions, which are more facile and more economical to obtain, are also ideal for the proposed application and very compatible with the matrix, easy to be dispersed and creates no particle agglomeration, which is a common phenomenon at composites with particles with dimension under 100 nm.

### 2.2. Equipment

#### 2.2.1. Processing Equipment

The composite materials were obtained by mechanically mixing the silicone rubber components with the ferrite powder in glassware specific to chemistry laboratories and pressing the obtained mixture in a press with hot plates.

#### 2.2.2. Characterization Equipment 

The characterization of these materials was carried out using the following equipment:

- Analytical balance type XS204, precision: 0.1 mg; linearity: ±0.2 mg; density kit for solids and liquids;
- Scanning electron microscope with field emission source and focused ion beam (SEM)—equipment dedicated to the study of microscopic structures and surfaces of different types of materials. There can be studied: inorganic samples and organic samples: polymers, plastics, polycomposite materials, electrically conductive or non-conductive materials, magnetic materials, as well as materials in compact form, powders, or thin layers. Images were taken at an accelerating voltage of 1 or 2 kV with very close proximity to the objective lens. The detector used was an Everhart Thorn-ley type secondary electron detector with a Faraday cup—resulting in micrographs highlighting the morphology and topography of the analyzed surfaces. The fields recorded in these micrographs are relatively narrow from a few hundred nm to microns, depending on the magnification used. Thus, SEM analyses were performed for all the composite materials obtained, as well as for the ferrite powder used.
- Dielectric spectroscopy (SOLARTRON Dielectric Spectrometer, Solartron Analytical, Farnborough, UK) and the impedance analyzer type 4294A (Agilent Technology, Santa Clara, CA, USA) and the kit for measuring magnetic properties 16454A;
- Signal Generator Rohde & Schwarz SMCV100B RF (Munich, Germany), min 4kHz, max 3GHz;
- Signal analyzer Rohde & Schwarz FPC1500 Desktop Spectrum Analyser (Munich, Germany), 5 kHz-3 GHz; Coaxial transmission device; Anechoic chamber; Schwartzbeck antennas (Schönau, Germany);
- The TG/DSC thermal analysis of composite plastic materials was performed on a STA 449 F3 Jupiter TG-DSC simultaneous thermal analyzer (NETZSCH, Selb, Germany). This analyzer allows the determination of mass variations and thermal changes for different types of materials.

### 2.3. Preparation Methods

#### 2.3.1. Obtaining Composite Materials

Obtaining the composite materials consisted of mixing and homogenizing the ferrite powder with preset percentages in the polymer mass, followed by the addition of catalyst and final homogenization towards composite. The obtained mixture was inserted into an open mold in a hot plate press at 60 °C for 1 h.

The definition of the obtained composite materials and their coding was presented in Table 2.

#### 2.3.2. Characterization of the Obtained Materials

The Determination of the Hydrostatic Density was Carried out According to [55,56]
SEM Structural Analyses were Carried out According to [57]
Tests to Determine Magnetic Permeability [58]

**Figure 1:**
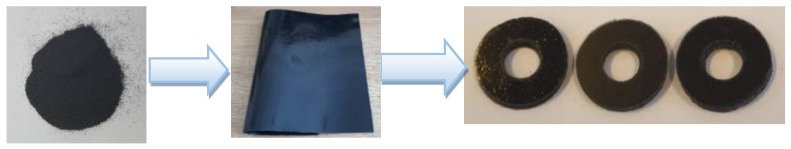
Samples processing to determine the magnetic permeability.

**Figure 2:**
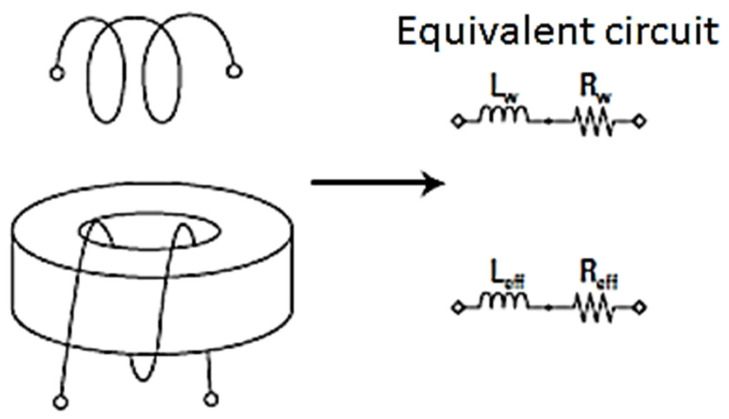
The equivalent measuring circuit.

To measure the magnetic permeability for such composites, an innovative procedure was applied, i.e., a predefined coil is wounded around the torus (as presented in Figure 1) and the inductance is measured at the ends of the wire. Permeability is derived from inductance measurements according to the following equations [38] and to Figure 2:

(1) $$\mu^{\prime }=\frac{lL_{eff}}{\mu_{0}N^{2}A}$$

(2) $$\mu^{\prime \prime }=\frac{l(R_{eff}-R_{w})}{\mu_{0}N^{2}\omega A}$$

where:

*R_eff_*: the equivalent resistance of the magnetic core losses including the wire resistance;
*L_eff_*: the inductance of the coil;
*N*: the number of windings;
*R_w_*: wire resistance;
*l*: average length of the magnetic core;
_A_: cross-sectional area of the magnetic core;
ω = 2πf (f—frequency)
μ_0_ = 4π 10^7^ (H/m)
Features of the measuring device:
Measured parameters: |Z|, |Y|, R, C, D, Q, ε*; µ*; tgδ
The measurement frequency: 40 Hz–110 MHz;
Configuration terminal: 7 mm
Compensation: open, short, and load

#### Thermal Analysis—Differential Scanning Calorimetry

TG-DSC (Differential Scanning Calorimetry) independently measures the heat flow rates to a sample and a reference, subjected to the same temperature program (isothermal or dynamic). The difference in heat flow between the sample and the reference, which are heated (or cooled) over a certain temperature range, is then determined and this difference is plotted as a function of temperature. The direction of the thermal flow towards the DSC sensor is well-defined and reproducible [59].

#### Broadband Dielectric Measurements

**Figure 3:**
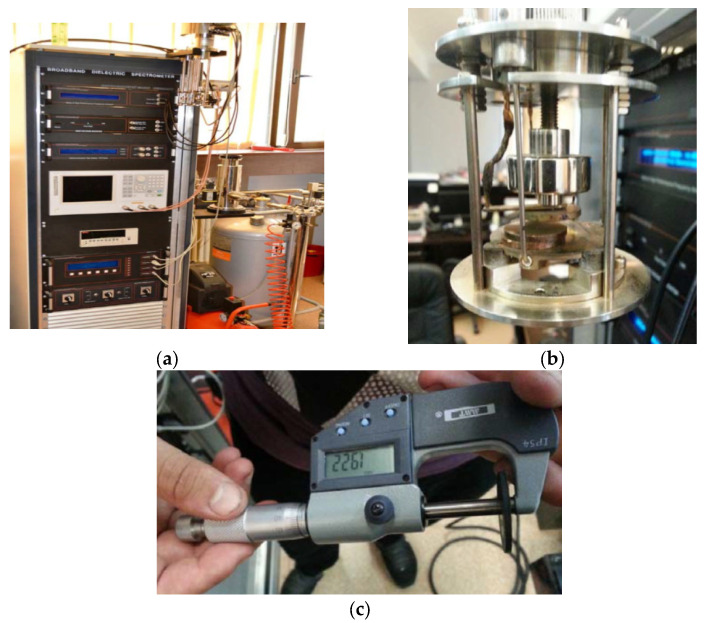
Measurement setup: (**a**) broadband dielectric spectrometer, (**b**) measurement cell, and (**c**) measurement of individual sample thickness.

Measurements were performed on the composite samples obtained in the laboratory, with the type of nanofillers mentioned before, Figure 3. Measurements (including those for the matrix only) were carried out using the Broadband Dielectric Spectrometer (Novocontrol GMBH) encompassing an Alpha frequency response analyzer and Quattro temperature controller (Figure 3a) with tailored measurement cells (as in Figure 3b). The manufactured samples were sandwiched between two copper electrodes of 20 mm diameter and placed inside the temperature-controlled cell (Figure 3b).

## 3. Results and Discussion

### 3.1. Determination of Hydrostatic Density

The determination of the hydrostatic density was carried out according to [55,56], and the obtained values are presented in Table 3. The obtained composite materials have densities in the range of 1.64–2.59 g/cm^3^, much lower than that of a metal material currently used as an electromagnetic screen (${\rho_{Cu}=8.96\;g/{cm}^{3},\rho }_{Fe}=7.87\;g/{cm}^{3}{,\rho }_{Ni}=8.91\;g/{cm}^{3}$).

### 3.2. SEM Structural Analyses

**Figure 4:**
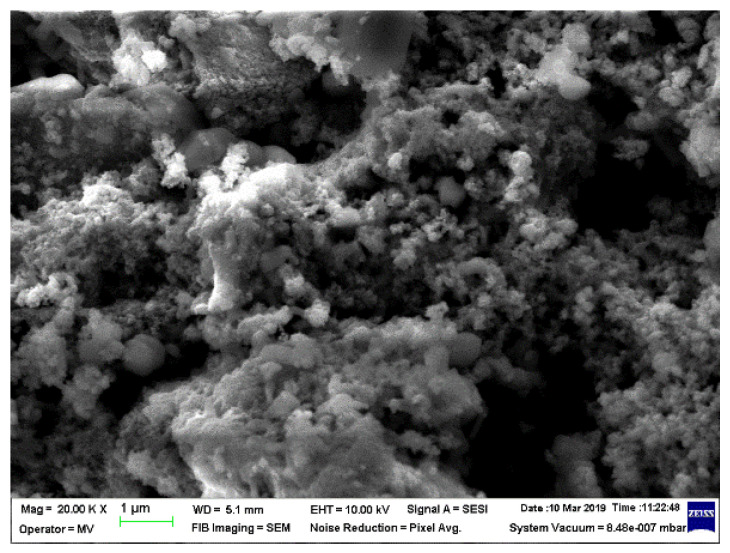
Micrographs of NiFe_2_O_4_ with magnification 20,000.

**Figure 5:**
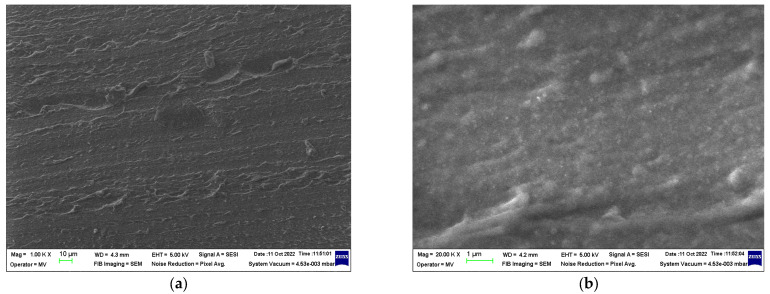
Micrographs of S0 material with magnifications (**a**) 1000× and (**b**) 20,000×.

**Figure 6:**
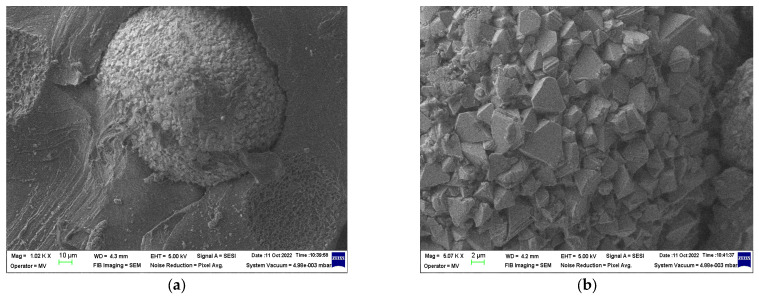

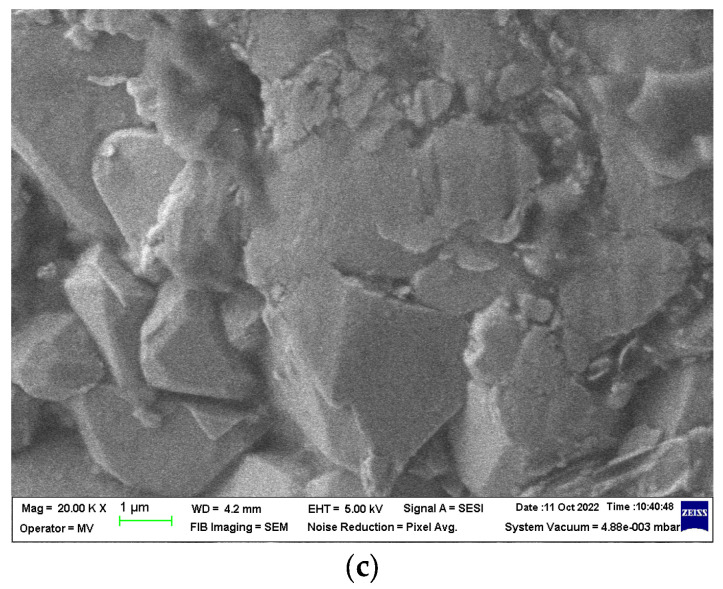
Micrographs of S1 material with magnifications (**a**) 1000×, (**b**) 5000×, and (**c**) 20,000×.

**Figure 7:**
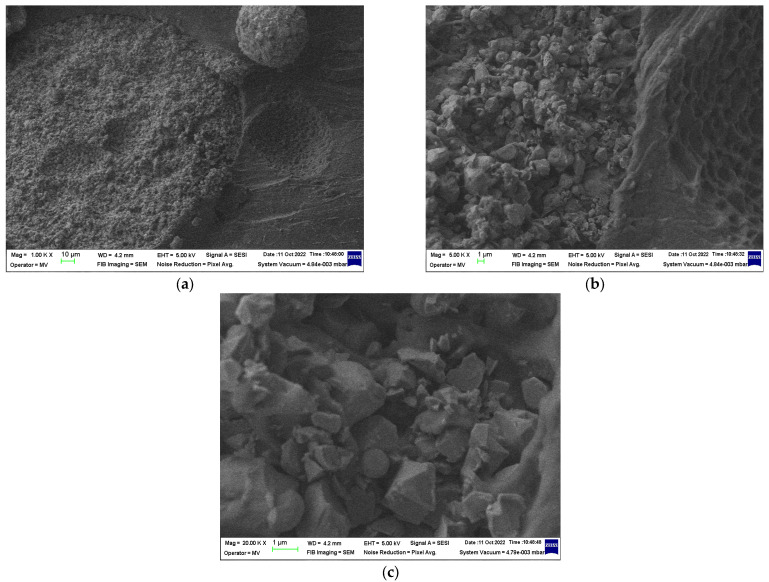
Micrographs of S2 material with magnifications (**a**) 1000×, (**b**) 5000×, and (**c**) 20,000×.

**Figure 8:**
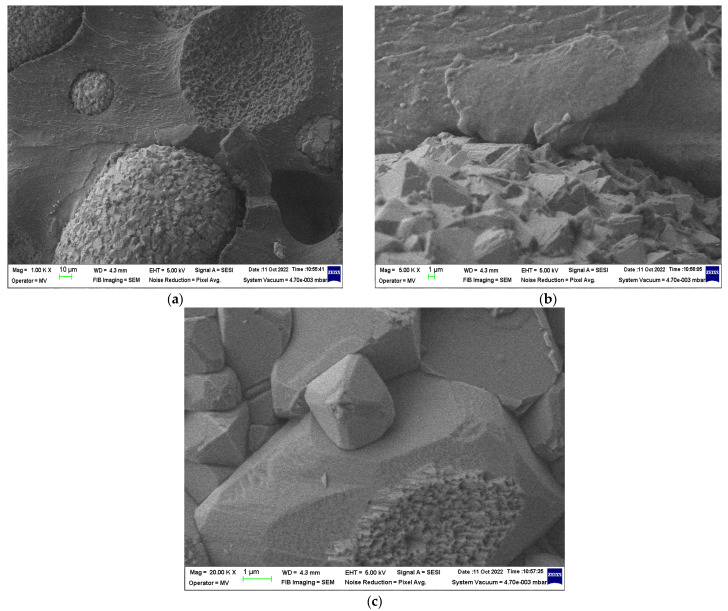
Micrographs of S3 material with magnifications (**a**) 1000×, (**b**) 5000×, and (c) 20,000×.

**Figure 9:**
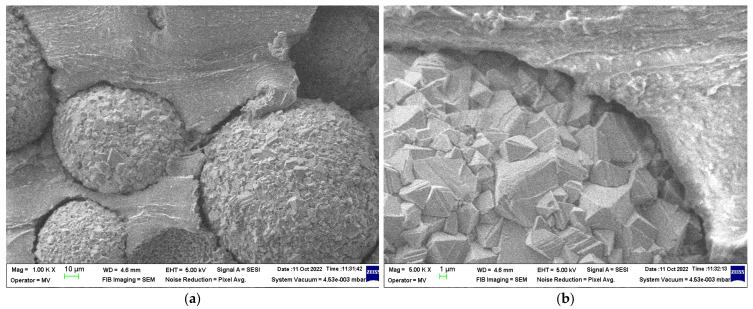

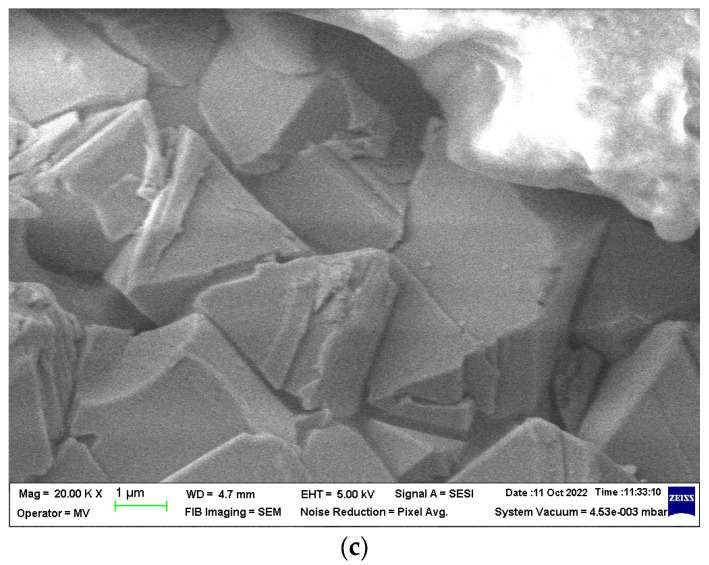
Micrographs of S4 material with magnifications (**a**) 1000×, (**b**) 5000×, and (**c**) 20,000×.

SEM structural analyses were performed to highlight the degree of homogeneity of the obtained materials. These analyses were performed on the field emission source and focused ion beam scanning electron microscope according to [57]. Figure 4, Figure 5, Figure 6, Figure 7, Figure 8 and Figure 9 show the micrographs for the NiFe_2_O_4_ powder and the S0–S4 coded samples at different magnifications.

From the micrographs made on these 5 experimental models, it can be seen that the filler is well-homogenized within the polymer structure with uniform dispersion. Though, slight agglomerations of the filler particles may appear, which induces a slight inhomogeneity aspect, however in small negligible areas, which can be remediated by a longer time mixing and homogenizing of the ferrite powder at higher powder content. 

### 3.3. Electromagnetic Tests

#### 3.3.1. Tests to Determine Magnetic Permeability

The tests to determine the magnetic permeability were carried out according to point “Tests to determine magnetic permeability” and from the values obtained, the efficiency of the electromagnetic shielding was determined.

The total electromagnetic shielding efficiency of 2 mm thick samples is given by the sum of reflection and absorption (3) [58]:

*SE* = *SE_R_* + *SE_A_* (3)

The formulas for reflection and absorption are (4) and (5), respectively [34]:

(4) $${SE}_{R}\left(dB\right)=10log(\frac{\sigma_{AC}}{16\omega \mu_{r}\varepsilon_{0}})$$

(5) $${SE}_{A}\left(dB\right)=20\frac{d}{\delta }\cdot \log e=20\cdot d\sqrt{\frac{\mu_{r}\omega \sigma_{AC}}{2}}\cdot \log e$$

where *d* is the thickness of the screen (in m), representing the depth of the radiation penetrates the material, (6):

(6) $$\delta =\sqrt{\frac{2}{\omega_{H}\mu \sigma_{AC}}}$$

where *σ* represents the material conductivity (7):

(7) $$\sigma =2\pi f\varepsilon_{0}\varepsilon_{r}tg\delta$$

By expanding (3), we obtain (8):

(8) $$SE\left(dB\right)\approx 10\log\left(\frac{\sigma }{16\omega \varepsilon }\right)+20\cdot d\sqrt{\frac{\mu_{r}\omega \sigma_{AC}}{2}}$$

Basically, in order to estimate the shielding efficiency, Formula (8) is used.

Magnetic tests were performed in the frequency range of 1–250 kHz. It is noted that the addition of ferrite powder in the polymer composition in percentages of 50, 60, 70, and 80% leads to an increase in SE from 16—in the case of the material with 0% ferrite (S0) to 32.25 in case of the material with 80% ferrite (S4). The μ values increase by increasing ferrite concentration. The experimental μ values obtained at different frequencies are presented in Table 4.

**Figure 10:**
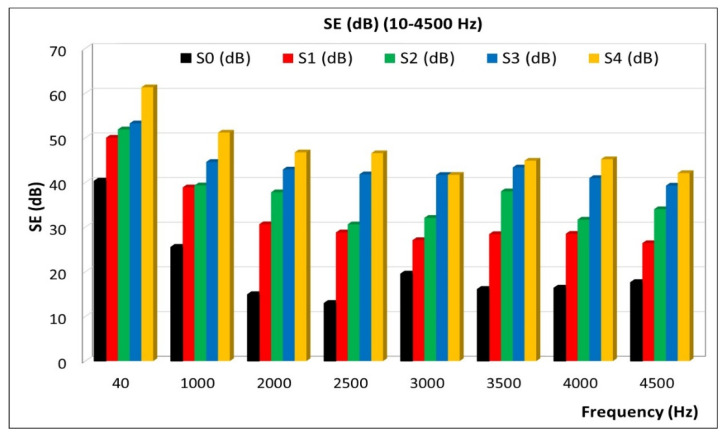
Variation of reflection at lower frequency.

These materials are recommended for use at low frequencies (up to 2000 Hz). These frequencies are dangerous for the living being. In Table 5 and, respectively, in Figure 10, the values of reflection are presented, for frequencies up to 4500 Hz.

The results presented above may suggest for S4 material also biomedical applications, where electromagnetic shielding at low frequencies is compulsory.

#### 3.3.2. Comparative Dielectric Tests

**Figure 11:**
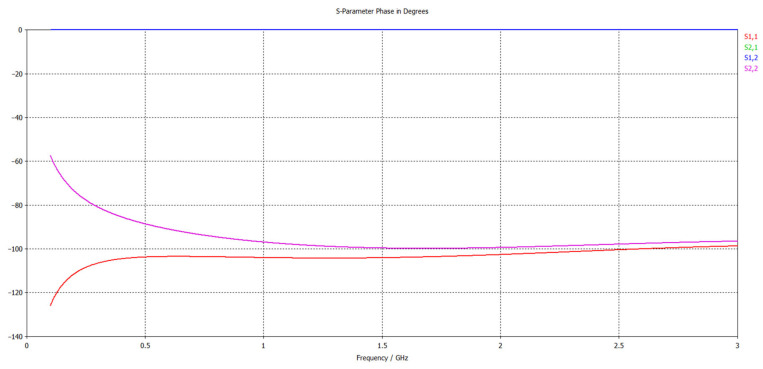

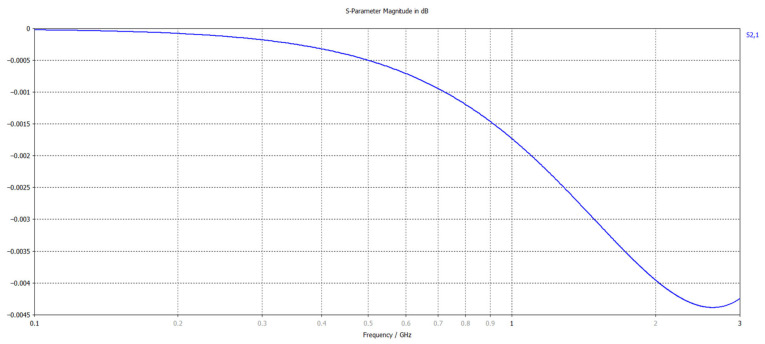
Variation of S and S21 parameters with frequency for composite materials coded as S2.

**Figure 12:**
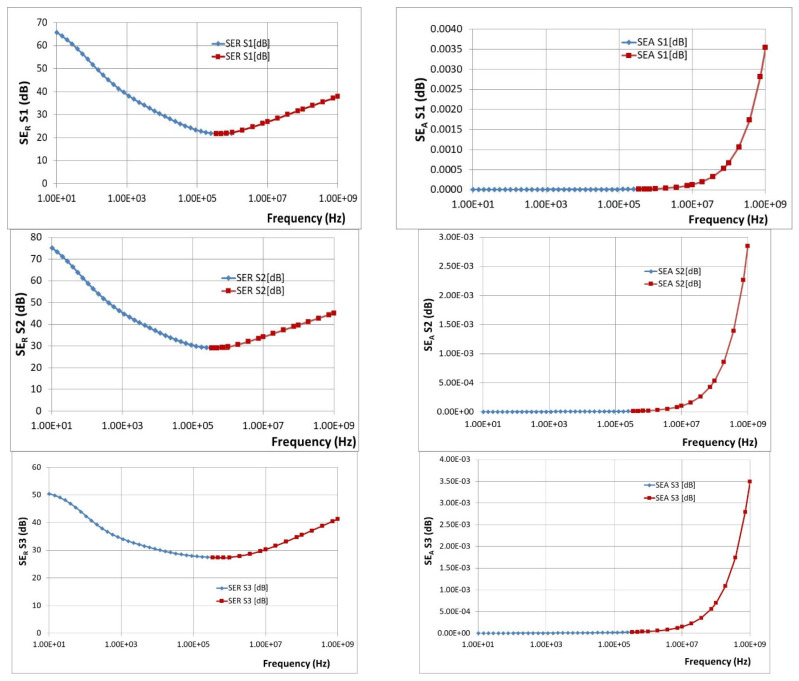

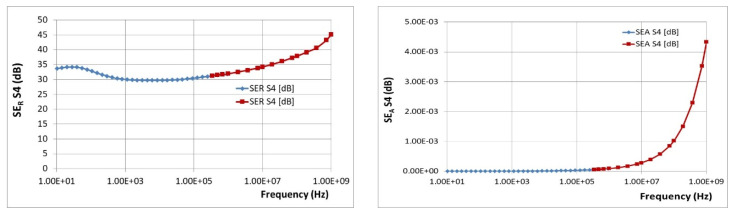
Variation of SE_R_ and SE_A_ with frequency for composite materials coded as S1–S4.

The preliminary dielectric test evolutions are briefly presented in Figure 11, as variations of parameters S. It is obvious that for GHz domain S21 parameter—absorption coefficient increases vs. frequency. The aggregated dielectric parameters are determined as described in “Broadband Dielectric Measurements”, by using 2 different cells for frequencies up to 10^6^ Hz, respectively, from 10^7^ Hz up to 10^9^ Hz (characteristics with blue, respectively, with red in Figure 12). The complete evolution of processed dielectric parameters was in concordance with the magnetic permeability variation. It is found that for these materials, at frequencies up to 10^6^ Hz, mainly reflection as shielding effect (i.e., relevant values for SE_R_) occurs, and respectively, absorption as the shielding effect is negligible. This can be explained by the fact that the shielding effect due to absorption (i.e., values of SE_A_) dramatically depends on the thickness of the samples, and our materials are relatively thin, i.e., in the range of 2.5–2.75 mm thickness. In this case, the contribution of interfacial polarization and collateral magnetic effect is detrimental to the reflectance features. At frequencies over 10^7^ Hz, the absorption as shielding effect progressively occurs and superposes itself over the reflection shielding effect, which diminishes but remains relevant. This aspect is explained by the internal architecture of the composite, i.e., the balance between the powder content and powder dimension, which is also correlated to the high increase in the conductivity at the GHz domain, even if the polarization effect is diminished. The study of such effects at the micro-scale and correlation with composition and architecture may be further studied as described in [60].

Corroborating the results from Figure 12, it can be said that SE_R_ is SE_A_ fall within the ranges presented in Table 6.

**Figure 13:**
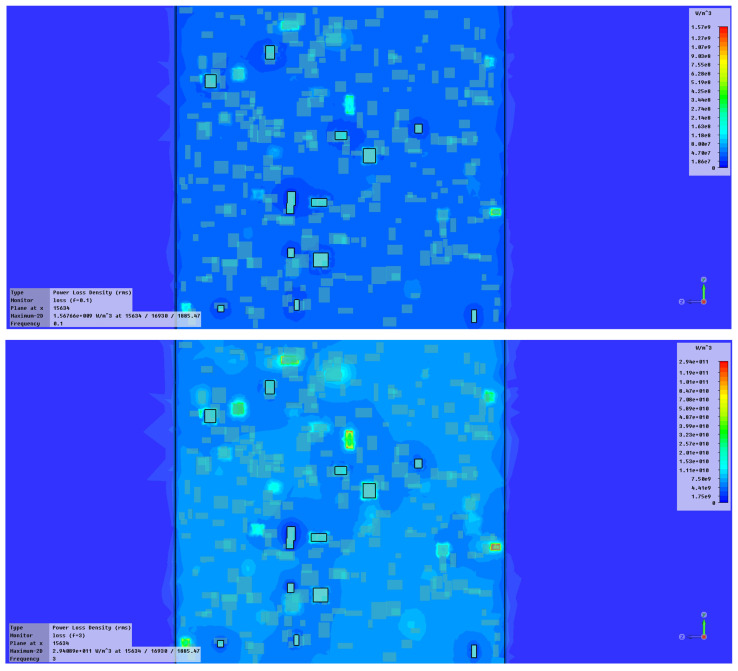
Power loss density in inhomogeneous composites with ferrites—sample S2, at 0.1 GHz and 3 GHz, respectively.

**Figure 14:**
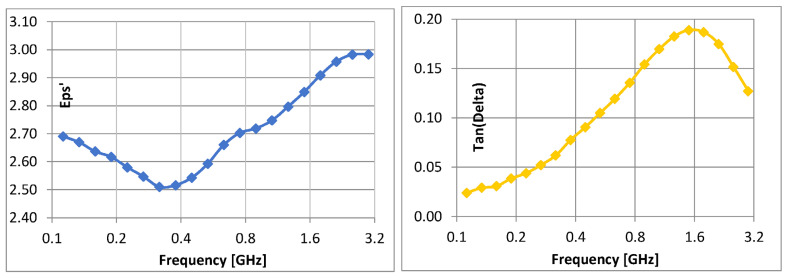
Dielectric parameters vs. frequency, GHz domain—sample S2.

Studying the SE behavior of composite materials analyzed at lower frequencies, S2 may provide the best electromagnetic shielding. At the microwave domain (around 1 GHz), the best characteristics are theoretically provided by S2 and S4, S4 having also a superior value of SE_A_. In principle, the electromagnetic shielding effect at lower frequencies represents the synergy between the results obtained by dielectric measurements and the results obtained by magnetic measurements, as presented in Figure 10. It is obvious that the composites with higher ferrite content, in our case S4, are ideal for electromagnetic shielding at lower frequencies. Surprisingly, the study emphasized also an important shielding effect at the GHz domain of composites with tailored architecture and lower ferrite content, in this case, S2. This aspect demonstrates the possibility of using nano-composites based on silicon and NiFe_2_O_4_ powders as shielding materials in the microwave domain, opening new opportunities for flexible devices for microwave technology. The development of broadband shielding composites for the microwave domain is a very new direction of research, and up to now only pure ceramic composites were studied, e.g., FeNi_3_/NiZnFe_2_O_4_/ZnO composite in [61], with clear drawbacks related to synthesis and final shaping technology, so ferrite-rubber composites, would be a good candidate for such applications, as our study emphasizes. Based on the theory from [60], a simulation is briefly presented in Figure 13—power loss density (proportional with SE_A_) in inhomogeneous composites with ferrites, here for sample S2, at 0.1 GHz and 3 GHz, respectively. The higher intensity of color for the 3 GHz diagram and activity of insertions can be noticed, along with the clear increase in absorbed power in Wm^−3^ compared to the diagram for 0.1 GHz. The conclusions are also in accordance with the dielectric parameters at the GHz domain, as presented in Figure 14, where a clear increase is emphasized, to be explained also based on the composite architecture and activity of ferrite—for the permittivity and on the conductivity increase—for dielectric loss, a parameter connected also with the absorbed energy. For the particular case of S2, the inhomogeneous composite presents a quasi-uniform dimension of ferrite powder and the space between ferrite particles in the composite has a fractional value of that dimension (about 20%), as occurring in Figure 7, an aspect that can be further correlated to the own frequency of the waveguide assimilated to S2 composite structure in the microwave domain, which presents a maximum activity at 3 GHz.

### 3.4. Thermal Stability

DSC measurements are recorded by thermal analyzer type STA 449 F3 Jupiter, Netzsch (Selb, Germany), working in the temperature range up to 1550 °C, in an inert, oxidizing, reducing, static or dynamic working atmosphere. The device is provided with a vacuum system with a maximum of 10^−2^ mbar [62].

**Figure 15:**
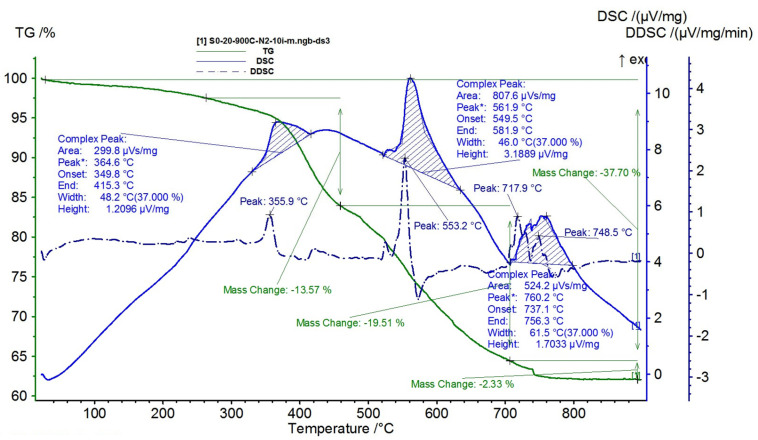
TG and DSC variation curves for sample S0—silicone rubber.

**Figure 16:**
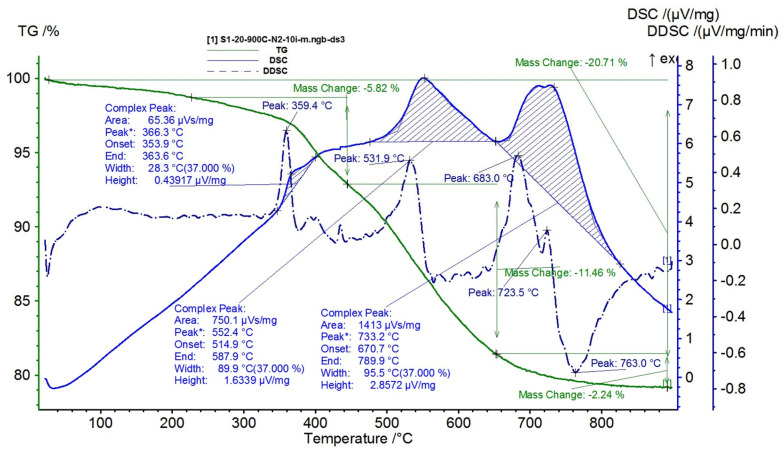
TG and DSC variation curves for sample S1—silicone rubber with 50% ferrite.

**Figure 17:**
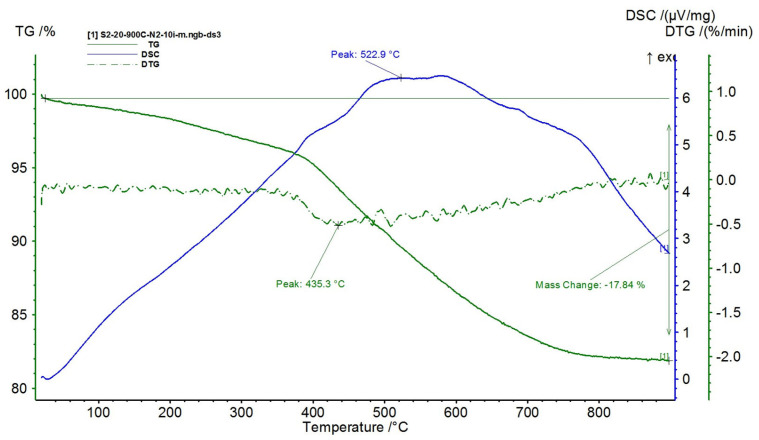
TG and DSC variation curves for sample S2—silicone rubber with 60% ferrite.

**Figure 18:**
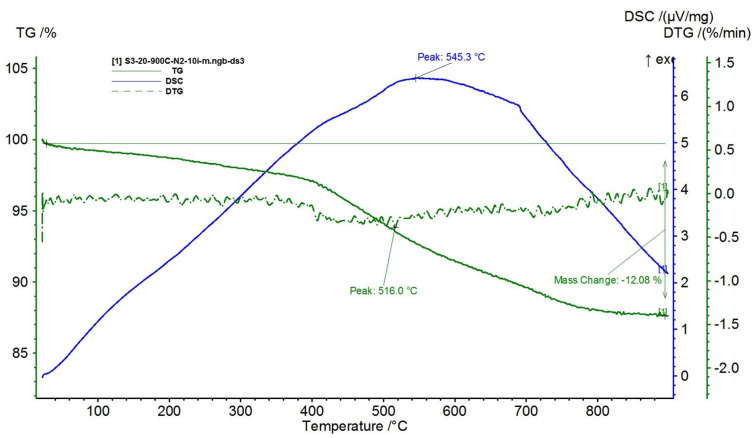
TG and DSC variation curves for sample S3—silicone rubber with 70% ferrite.

**Figure 19:**
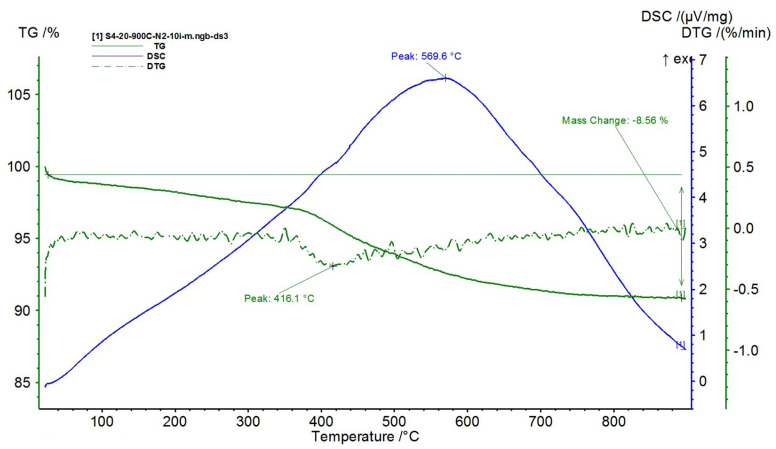
TG and DSC variation curves for sample S4—Silicone rubber with 80% ferrite.

The results are presented in Figure 15, Figure 16, Figure 17, Figure 18 and Figure 19. 

From the TG-DSC thermal analysis performed for silicone rubber, Figure 15 it is observed that, once it is heated at 10 k/min, in a gaseous environment (N_2_), 3 stages of degradation are identified:

- The first degradation stage takes place at the maximum temperature of 364.6 °C, which corresponds to a mass loss of 13.57%;
- The second degradation stage has a higher intensity and occurs at the maximum temperature of 561.9 °C, corresponding to a mass loss of 19.51%;
- The third degradation stage takes place at the maximum temperature of 760.2 °C and shows a small mass loss of 2.33%.

The total mass loss recorded for silicone rubber, over the entire temperature range, is 37.70%.

By introducing NiFe_2_O_4_ into the silicone rubber, a composite is formed whose behavior during thermal analysis varies depending on the added NiFe_2_O_4_ content (50, 60, 70, and 80%, respectively).

At equal proportions of 50% of NiFe_2_O_4_ and silicone rubber, Figure 16, the three stages of degradation are preserved, as follows:

- The first degradation stage takes place at the maximum temperature of 366.3 °C, which corresponds to a mass loss of 5.82%;
- The second degradation stage occurs at the maximum temperature of 552.4 °C, corresponding to a mass loss of 11.46%;
- The third degradation stage takes place at the maximum temperature of 733.2 °C and presents a mass loss of 2.24%.

For the entire range analyzed (20–900 °C), the total mass loss for the composite with 50% Ni ferrite and 50% silicone rubber was 20.71%.

With the increase in the proportion of NiFe_2_O_4_ in the composite (over 60%), in Figure 17, Figure 18 and Figure 19, the three decomposition processes are suppressed, highlighting only one decomposition process over the entire analyzed temperature range.

Furthermore, the decomposition temperature increases with the increase in the NiFe_2_O_4_ content in the composite and the mass losses decrease, i.e.:

- For the composite with 60% NiFe_2_O_4_, a decomposition temperature of 522.9 °C was recorded, reaching a total mass loss of 17.84%;
- For the composite with 70% NiFe_2_O_4_, a decomposition temperature of 545.3 °C was recorded, reaching a total mass loss of 12.08%;
- For the composite with 80% NiFe_2_O_4_, a decomposition temperature of 569.6 °C was recorded, reaching a total mass loss of 8.56%.

**Figure 20:**
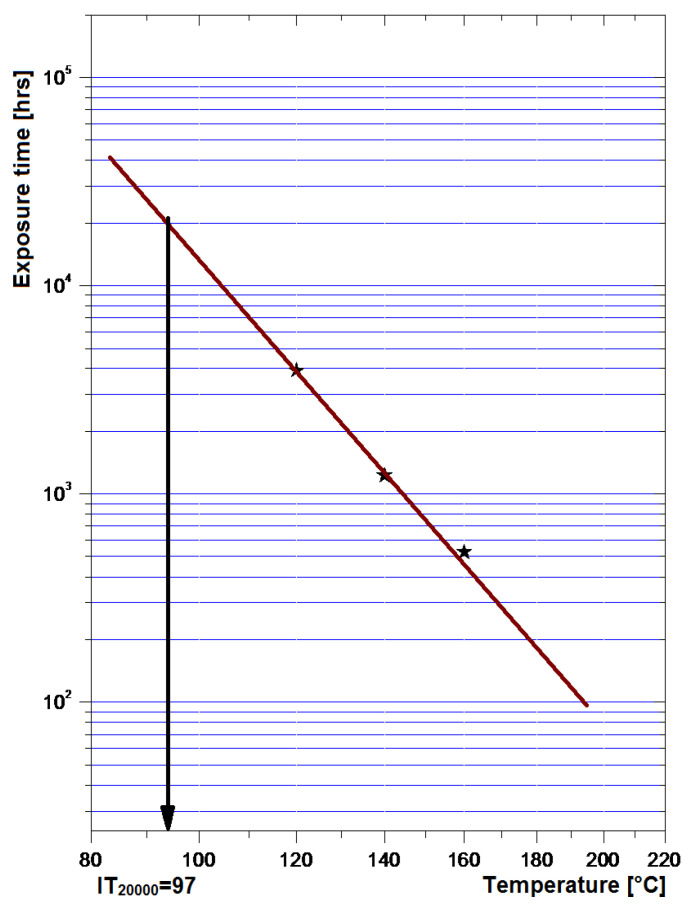
Extrapolation curve for lifetime evaluation—S4.

Following the TG-DSC tests, the usage temperature supported by the analyzed composites will be lower than 360 °C, because at this temperature the first thermal degradation of the polymer matrix occurs. Finally, based on thermal analysis results, thermal endurance was established, in order to approximate the relationship between the lifetime of the material and temperature, the temperature superior limit being within the interval of the first degradation stage. As composites from silicone rubber do not present a melting temperature point or interval at elevated temperatures, and they became solid and brittle at elevated temperatures, in the case of lifetime evaluation we preferred a thermal exposure under 250 °C at which practically the composite features are not altered, so a temperature much lower than the temperature of the first degradation, but enough for lead to reasonable temporal data. The degradation criterion was considered the mass loss, i.e., it is considered that the end of life of the composite material at different temperatures corresponds to a 10% mass loss. The extrapolation curve is presented in Figure 20, in order to identify the exploitation temperature limit for which the service duration is over 20,000 hrs, the minimum imposed for electric/electronic applications. The achieved result is very promising, with the service temperature within the limit of 20,000 hrs. being 97 °C, much higher compared to the requested 70 °C for electric/electronic applications. Hence, the respective composites are very stable in temperature, provide a much higher lifetime as requested, and can be exploited at higher temperatures, i.e., can expand the applications for the microwave domain where the absorbed energy represents an important aging factor.

### 3.5. Flexibility Tests

There is no standard method related to the test of flexibility for this type of rubber composite, and consequently, in this paper, a demonstrative functional test is presented, in order to assess the sample’s flexibility, in line with the method presented in [63].

**Figure 21:**
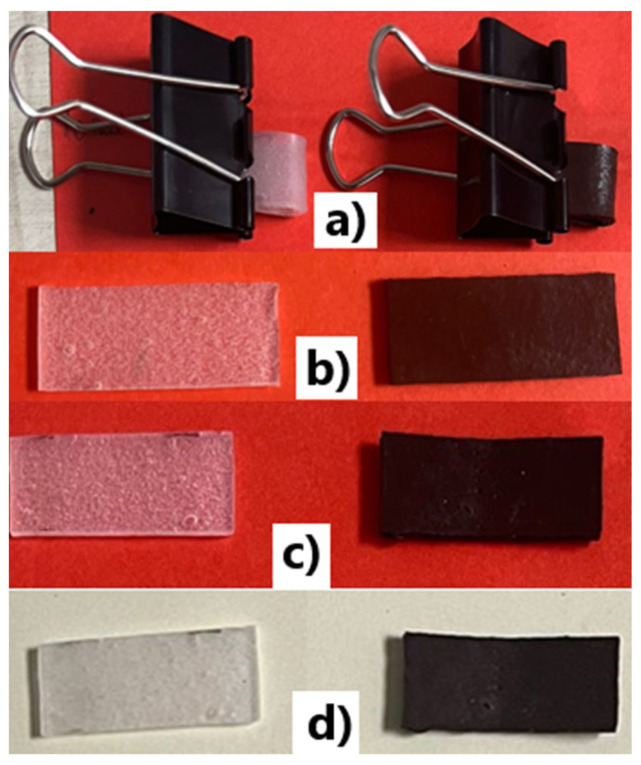
Functional flexibility test for S0 and S2 samples: (**a**) initial state, (**b**) the state after 30 min. fixation and 10 min. relaxation, (**c**) the state after 1 h fixation and 10min. relaxation, (**d**) the state after 30 h. fixation and 10 min. relaxation.

The test was made comparatively upon samples from silicone rubber without added ferrite (S0) and with the highest percentage of ferrite (S2, 80% ferrite). Taking into account the most demanding functional stress, samples with minimal dimensions of 35 mm × 15 mm × 2.5 mm were cut and clamped, and fixed with dovetail clips as shown in Figure 21 for different periods, i.e., 30 min, 1 h, and 30 h, and then are released and observed after 10 min. relaxation time. In all tests, no exfoliation or cleavage was noticed. S0 practically reached the initial shape after 10 min. relaxation period for all stress periods. S8 reached the initial shape after 10 min. relaxation period for a stress of 30min, but also in the case of the other stress periods the shape recovery was fairly well after 10 min. relaxation, and practically reached after about 1 h relaxation. In all, for both sample types, the relaxation effect is similar, which means that such rubber composites exhibited good flexibility.

Finally, the test of samples hardness was performed, by use of a common Shore D mechanical hardness tester for flexible materials (as rubber and plastic samples) [64], with a scale from 0 to 100 units, and a 30° point test tip. The results are presented in Table 7.

The results partially explain the flexibility and elastic behavior of rubber composites, the maximum value is 25, related to “under medium—hard” domain. 

## 4. Conclusions

Composite materials with silicone rubber as polymer matrix and ferrites have the great advantage of being very flexible, and able to be attached to any architecture of electronic or automotive device in order to perform shielding properties, including electromagnetic shielding.

In this work, five types of composite materials with silicon as polymer matrix and nickel ferrite powders at different concentrations (up to 80) were obtained and characterized, in order to potentially become successful candidates as flexible electromagnetic shielding materials for automotive, biomedical, or wearable electronics applications. NiFe_2_O_4_, a soft super-paramagnetic material with magnetic semiconductor features, was peculiarly chosen for the purpose of avoiding the supplementary addition of conductive powders, a procedure commonly used to improve the ferrite behavior under the electromagnetic field. 

The ferrite powder was synthesized by a simple and innovative co-precipitation route, having an average dimension of about 750 nm. The procedure is much simpler and economical compared to similar ones, such as co-precipitation with mechanical alloying, or sol–gel auto-combustion, and sub-micron particles are easier to be dispersed and created no particle agglomeration within a composite structure. Beyond the well-known advantages of using silicon matrix for composites, another important advantage lies in the possibility of making multi-layer structures or linked structures, including by use of common commercial silicon adhesives, aspects very important in electronic technology, which maintain the flexibility of the structure, advantage not met by other types of rubbers, where no compatible adhesives are available to maintain both flexibility and strength. 

The composite materials were obtained by mixing and homogenizing the ferrite powder with preset percentages in the polymer mass, followed by the addition of catalyst and final homogenization, and inserted into an open mold in a hot plate press at 60 °C for 1 h. From the micrographs, it can be seen that the filler is well-homogenized within the polymer structure with uniform dispersion. 

As regards electromagnetic field shielding, it was found that for these materials, at frequencies up to 10^6^ Hz, mainly reflection occurs as a shielding effect. This can be explained by the fact that the shielding effect due to absorption dramatically depends on the thickness of the samples, and our materials are relatively thin, i.e., in the range of 2.5–2.75 mm thickness. The composite material with 80% ferrite powder represents the most successful material to be used for electromagnetic shielding at low-frequency domains, where also biomedical applications can be foreseen. 

At frequencies over 10^7^ Hz, the absorption as shielding effect progressively occurs and superposes itself over the reflection shielding effect, which diminishes but remains relevant. This aspect is explained by the internal architecture of the composite, i.e., the balance between the powder content and powder dimension, which is also correlated to the high increase in the conductivity at the GHz domain, even if the polarization effect is diminished. The observations are in accordance with the dielectric parameters al GHz domain, where a clear increase is emphasized, to be explained also based on the composite architecture and activity of ferrite—for the permittivity and on the conductivity increase—for dielectric loss, a parameter connected also with the absorbed energy. The study emphasized an important shielding effect at the GHz domain of the sample with 60% ferrite content, an aspect that can demonstrate the possibility of using the composites for applications in the microwave domain, opening new opportunities for flexible devices for microwave technology. The flexibility of samples was emphasized by a demonstrative functional test, which revealed that all samples practically reached the initial shape after 10 min relaxation period for all stress periods up to 30 h.

From the TG-DSC thermal analysis, it was observed that the first degradation stage takes place at a maximum temperature of about 400 °C, which corresponds to a mass loss of about 15%. As composites from silicone rubber do not present a melting temperature point or interval at elevated temperatures, and they became solid and brittle at elevated temperatures, in the case of lifetime evaluation, we preferred a thermal exposure under 250 °C, at which practically the compo-site features are not altered. The achieved result is very promising, the service temperature is within the limit of 20,000 h. being 97 °C, much higher comparing to the requested 70 °C for electric/electronic applications. Hence, the respective composites are very stable in temperature, provide a much higher lifetime as requested, and can be exploited at higher temperatures, i.e., can expand the applications for the microwave domain where the absorbed energy represents an important aging factor. 

## Figures and Tables

**Table 1:** HT 45 polymer properties.

Property	Value
Hardness—Shore A (after 24 h)	43 ± 3
A/B Mix Ratio (weight parts)	100/100
Reproduction detail (micron)	2
Mixing time/minutes, at 23 °C	2′
Working time/minutes, at 23 °C	10′–12′
Total demolding time, at 23 °C (hours)	1–1 h 30′
Dimensional variation (after 24 h)	0.05%
Mixture viscosity—centipoise (cP)	4000 ± 300
Elongation to break—%	370 ± 20%
Breaking strength (DIE B—N/mm)	3.5 ± 0.5

**Table 2:** Sample composition and coding.

Composite Material	Composition (%) HT45/NiFe_2_O_4_
S0	100/0
S1	50/50
S2	40/60
S3	30/70
S4	20/80

**Table 3:** The hydrostatic density of the studied materials.

Encoding	S0	S1	S2	S3	S4
The density (g/cm^3^)	1.004	1.642	1.924	2.150	2.594

**Table 4:** The magnetic permeability of the materials under study.

Frequency (Hz)		S0	S1	S2	S3	S4
	Magnetic Permeability (μ)					
0		1.99E+04	2.24E+04	9.83E+03	1.58E+04	3.71E+04
1000		1.17E+01	7.79E+02	8.54E+02	1.52E+03	3.15E+03
5000		9.85E−01	3.84E+01	8.01E+01	5.99E+02	9.56E+02
10,000		8.99E−01	1.16E+02	8.00E+01	1.79E+02	3.78E+01
50,000		1.32E+00	1.71E+01	8.37E+00	7.30E+00	1.28E+01
100,000		1.28E+00	9.72E+00	7.08E+00	2.43E+00	1.84E+01

**Table 5:** Total SE (dB) up to 4500 Hz.

**Frequency (Hz)**	**S0 (dB)**	**S1 (dB)**	**S2 (dB)**	**S3 (dB)**	**S4 (dB)**
10	15.82	50.01	51.86	53.21	61.30
1000	15.86	38.89	39.32	44.59	51.15
2000	15.88	30.64	37.79	42.91	46.73
**Average up to 2000 Hz**	**16**	**40**	**43**	**47**	**53**
2500	15.90	28.80	30.61	41.82	46.53
3000	15.91	27.08	32.05	41.67	41.70
3500	15.92	28.42	38.01	43.35	44.86
4000	15.92	28.50	31.67	40.97	45.20
4500	15.93	26.41	34.01	39.28	42.08
**Average up to 4500 Hz**	**16**	**32**	**37**	**43**	**47**

**Table 6:** Comparative average values of SE_R_ and SE_A_.

Composite Material	Frequency Range (Hz)					
	10^2^ Hz		10^6^ Hz		10^9^ Hz	
	SE_R_	SE_A_	SE_R_	SE_A_	SE_R_	SE_A_
S0	8	0	12	6 × 10^−4^	24.5	0.08
S1	52	0	22	10^−4^	39	0.0035
S2	60	0	30	10^−4^	46	0.0029
S3	41	0	28.5	10^−4^	41	0.0035
S4	34	0	32.5	3 × 10^−4^	46	0.0044

**Table 7:** Hardness test values.

Sample Code	Hardness Value
S1	15
S2	17
S3	20
S4	25

## Data Availability

The data presented in this study are available on request from the corresponding author.
